# Tardive Dyskinesia in Patients Receiving Continuous Versus Intermittent Oral Metoclopramide: A Retrospective Real World Study

**DOI:** 10.1111/nmo.70422

**Published:** 2026-09-06

**Authors:** Pierantonio Russo, Ramaa Nathan, Michael Camilleri

**Affiliations:** ^1^ EVERSANA Life Sciences Overland Park Kansas USA; ^2^ Mayo Clinic Rochester Minnesota USA

**Keywords:** adverse effects, dopamine antagonist, gastroparesis, metoclopramide, tardive dyskinesia

## Abstract

**Background:**

Tardive dyskinesia (TD) is an often irreversible movement disorder associated with prolonged exposure to dopamine receptor antagonists such as metoclopramide (MCP). This study evaluates the prevalence and timing of TD in patients receiving continuous versus intermittent MCP.

**Methods:**

A retrospective analysis was conducted using Symphony Health Solutions data from July 2018 to June 2024, covering over 307 million U.S. patients. Two cohorts were analyzed: (1) all patients with ≥ 2 MCP prescriptions (Cohort A), and (2) patients with a gastroparesis diagnosis (ICD10 K31.84) prior to ≥ 2 MCP prescriptions (Cohort B). TD prevalence was evaluated based on ≥ 1 or ≥ 2 ICD‐10 G24.01 claims within 12 months after initial MCP prescription. MCP exposure was classified as continuous (Proportion of days covered (PDC) > = 1) or intermittent (PDC < 1).

**Results:**

TD prevalence was higher in patients receiving continuous MCP across both cohorts. In Cohort A (*N* = 1,360,196), TD prevalence was 0.25% (continuous) vs. 0.18% (intermittent) for ≥ 1 claim, and 0.13% vs. 0.09% for ≥ 2 claims. In the cohort with gastroparesis (*N* = 125,279), TD prevalence was 0.77% (continuous) vs. 0.55% (intermittent) for ≥ 1 claim, and 0.43% vs. 0.25% for ≥ 2 claims. Median time to TD diagnosis was shorter for continuous users: 567 vs. 758 days in Cohort A and 490 vs. 573 days in Cohort B. All group differences were statistically significant (*p* < 0.005).

**Conclusions:**

Continuous MCP dosing was associated with higher TD prevalence and earlier onset. Intermittent dosing, despite longer cumulative exposure, was associated with lower TD risk. These findings suggest that intermittent regimens may offer a safer MCP therapeutic strategy.

## Key Points

- Intermittent MCP doising is associated with lower TD risk.

## Plain Language Summary

Metoclopramide is the only medication approved in the U.S. to treat gastroparesis, a condition that slows stomach emptying. However, long‐term use of this drug can lead to tardive dyskinesia, a movement disorder that may be permanent. This study looked at over 1.36 million people who received metoclopramide to understand whether the way the medication is taken—continuously or with breaks—affects the risk of developing tardive dyskinesia. We found that people who took metoclopramide continuously had a higher risk of tardive dyskinesia than those who took it intermittently, even though intermittent users were treated for a longer overall period. Continuous users also developed symptoms sooner. These patterns were seen both in the general population and among patients with gastroparesis. These findings suggest that taking metoclopramide with planned breaks, rather than continuously, may lower the risk of this serious side effect. This may help guide safer long‐term treatment strategies.

## Graphical abstract

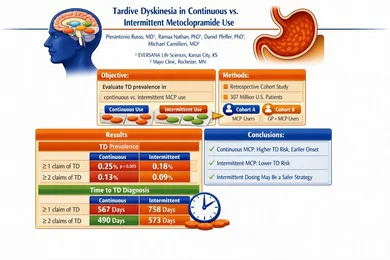

## 1 Introduction

Tardive dyskinesia (TD) is a debilitating, often irreversible movement disorder linked to chronic exposure to dopamine receptor antagonists [1, 2, 3]. Metoclopramide (MCP), a commonly prescribed agent for gastroparesis (GP), which is endorsed by ACG [4] and AGA guidelines on gastroparesis [5], carries an FDA boxed warning due to the TD risk associated with long‐term use [6]. Estimates of TD incidence vary. However, a comprehensive analysis of reports from 1984 to 2018 based on different cohorts ranging from 51 Veterans Affairs patients to national prescription databases in Scandinavia and United Kingdom or patient databases of 2 to 80 thousand patients concluded that the risk of TD from metoclopramide is in the range of 0.1% per 1000 patient years [7]. Similarly, analysis of the literature from 1984 to 2008 concluded that risk of TD from MCP use is likely to be < 1% [8].

Despite these risks inferred by the black box warning which has been established in 2009, MCP remains the only FDA‐approved treatment for GP, leading to widespread chronic use [4, 9]. While the relationship between dose duration and TD is implied by the FDA recommendation that the medication should not be used for > 3 months, the impact of different dosing patterns—specifically intermittent versus continuous MCP use—on TD risk has not been well studied [10].

This retrospective study aimed to quantify the prevalence and to assess the timing of TD in patients receiving oral MCP, stratified by continuous compared to intermittent use—a distinction particularly relevant given the well‐established dose‐ and time‐dependent risk of TD extending beyond standard 12‐week treatment restrictions, which provided the rationale for the FDA boxed recommendation. The study also evaluated the risk for the general population receiving MCP compared to those with a prior GP diagnosis who received oral MCP.

## 2 Materials and Methods

We conducted a retrospective observational study using administrative medical claims data extracted from Symphony Health Solutions' Integrated Dataverse, spanning July 2018 through June 2024. The dataset included prescription and diagnosis claims from > 50 billion transactions across approximately 307 million unique U.S. patients.

Two cohorts were analyzed: **Cohort A**: All patients with ≥ 2 oral MCP prescriptions and **Cohort B**: Subset of patients with a GP diagnosis (ICD10 K31.84) prior to receiving ≥ 2 oral MCP prescriptions.

All patients were required to have ≥ 12 months of continuous data available following their initial MCP prescription.

Treatment adherence for each MCP patient was assessed using the Proportion of Days Covered (PDC), calculated as the total days of medication possession (as specified by the days supply on each prescription claim) divided by the total duration from the first MCP prescription start date to the last prescription end date. Given the well‐established dose and time dependent nature of tardive dyskinesia—with risk extending well beyond the standard 12‐week treatment restriction—and the absence of electronic medical records documenting when patients were specifically advised to discontinue medication, prescription possession patterns were used to approximate real‐world treatment behavior. Patients in each cohort with a PDC of ≥ 1 were classified as “Continuous Use,” indicating uninterrupted prescription possession from treatment initiation through the last prescription end date with no gaps exceeding 7 days. Patients with a PDCbelow this threshold, reflecting gaps of more than 7 days between prescriptions, were classified as “Intermittent Use.”

TD was identified using ICD‐10 code G24.01 (drug‐induced subacute dyskinesia). To account for the time‐dependent emergence of TD, all patients were required to have a minimum of 12 months of follow‐up data following MCP initiation. Prevalence of TD was measured in two ways: first, with a minimal requirement of at least one claim for TD within 12 months of MCP initiation and second, with a stricter requirement intended to confirm diagnosis reliability, of at least two claims for TD within 12 months of MCP initiation.

### 2.1 Statistical Analysis

Chi‐squared tests were used to compare TD prevalence between groups: continuous vs. intermittent use for cohort A and B. Median time to TD diagnosis and total duration of MCP use were also evaluated. A *p*‐value < 0.005 was considered statistically significant (to account for the multiplicity of statistical comparisons).

## 3 Results

Cohort A comprised 1,360,196 patients who received oral MCP, with 10% categorized as continuous users. Cohort B included 125,279 patients diagnosed with gastroparesis prior to initiating MCP, of whom 8% were continuous users.

Among general MCP users in Cohort A (Table 1), the prevalence of TD based on at least one ICD documentation of TD was 0.25% in the continuous group compared to 0.18% in the intermittent group (*p* < 0.005). When a stricter definition requiring at least two TD claims was applied, prevalence dropped to 0.13% for continuous users and 0.09% for intermittent users (*p* < 0.005).

**TABLE 1:** Cohort A (#Patients = 1,360,196)—Prevalence of TD after initiation of MCP in patients with at least two oral MCP prescriptions.

Minimum Claims for TD	Continuous Use of MCP #Patients = 140,192	Intermittent Use of MCP #Patients = 1,220,004	*p*‐value
> = 1 claim for TD (#Patients = 2544)	345 (0.25%)	2199 (0.18%)	*p* < 0.005
> = 2 claims for TD (#Patients = 1294)	187 (0.13%)	1107 (0.09%)	*p* < 0.005

In Cohort B (Table 2), which consisted of gastroparesis patients, TD prevalence was notably higher. Continuous users had a 0.77% prevalence based on at least one claim, versus 0.55% in the intermittent group. When two claims were required for TD confirmation, the prevalence was 0.43% for continuous users and 0.25% for intermittent users.

**TABLE 2:** Cohort B (#Patients = 125,279)—Prevalence of TD after initiation of MCP in patients with Gastroparesis and at least two oral MCP prescriptions.

Minimum Claims for TD	Continuous Use of MCP #Patients = 9851	Intermittent Use of MCP #Patients = 115,428	*p*‐value
> = 1 claim for TD (#Patients = 707)	76 (0.77%)	631 (0.55%)	*p* < 0.005
> = 2 claims for TD (#Patients = 332)	42 (0.43%)	290 (0.25%)	*p* < 0.005

Time to TD diagnosis after onset of MCP therapy also differed by dosing pattern. In Cohort A, continuous users developed TD after a median of 567 days, while intermittent users developed TD later, after a median of 758 days. A similar pattern was observed in Cohort B, where continuous users developed TD after 490 days compared to 573 days in the intermittent group.

Although intermittent users had lower TD rates, they tended to remain on MCP longer than continuous users. In Cohort A, median treatment duration was 728 days for intermittent users and 567 days for continuous users. In Cohort B, intermittent users were on therapy for 573 days, compared to 490 days for those on continuous MCP regimens. All group differences were statistically significant, with *p*‐values less than 0.005.

## 4 Discussion

In this large, real‐world analysis of > 1.36 million MCP recipients (including 125,279 with GP), continuous oral MCP use was associated with both higher incidence of tardive dyskinesia (TD) and earlier onset than intermittent use. Across both the overall MCP cohort and the GP subcohort, relative risks for TD ranged from ~1.39 to 1.72 for continuous vs. intermittent exposure, and median time to TD diagnosis was shorter among continuous users by ~3–9 months. Importantly, these differences emerged despite longer observed treatment duration among intermittent users, suggesting that dosing pattern, not merely exposure time, may be a determinant of risk. Using the more specific definition of ≥ 2‐claims (documentations) of TD by ICD code, the absolute risk increase was ~0.04% in the overall cohort (number‐needed‐to‐harm ≈2500) and ~0.18% in the GP cohort (≈556).

TD is thought to arise from chronic dopaminergic D2 receptor blockade in the nigrostriatal pathway with downstream dopamine super sensitivity, maladaptive synaptic plasticity, and oxidative stress [1, 2, 3]. Intermittent MCP dosing may reduce sustained D2 occupancy, allow receptor resensitization, or lower cumulative neurotoxic stress, thereby attenuating risk compared with uninterrupted exposure.

The FDA's boxed warning for MCP emphasizes that TD risk rises with duration of treatment and total cumulative dose and advises avoiding therapy beyond 12 weeks across all formulations [6, 11]. The 2022 American College of Gastroenterology (ACG) guideline and AGA guideline of 2025 highlight MCP as the only FDA‐approved medication for GP and acknowledges the FDA boxed‐warning constraint of 3 months. Furthermore, both guidelines recommend using the lowest effective dose for the shortest duration, prioritizing safety monitoring [4]. The AGA guideline also suggests using 1–4 weeks drug holidays every 8–12 weeks in patients with gastroparesis, which is a disease that may affect patients for years.

The available evidence from systematic reviews and meta‐analyses (Table 3) [12, 13, 14, 15, 16] consistently indicates that TD remains a clinically important complication of antipsychotic therapy and that its risk is lower, though not abolished, with second‐generation antipsychotics (SGAs) than with first‐generation antipsychotics (FGAs). In the largest cross‐sectional synthesis (41 studies; 11,493 patients), the global mean TD prevalence was 25.3% (95% CI, 22.7–28.1), with current FGA treatment carrying a significantly higher prevalence than current SGA treatment (30.0% vs. 20.7%; *p* = 0.002); a pooled estimate from observational studies was lower at 7% (95% CI, 4–9), reflecting differences in sampling and ascertainment. Comparative randomized data reinforce this pattern: across 57 head‐to‐head trials, the annualized incidence of TD was 6.5% with FGAs versus 2.6% with SGAs (RR, 0.47; NNT, 20), with olanzapine and aripiprazole showing an advantage over other non‐clozapine SGAs. The risk differential is most pronounced in older adults, in whom the 1‐year incidence of probable TD was more than three‐fold higher with FGAs than SGAs (23% vs. 7%) and FGA‐associated cumulative incidence reached 57% by 3 years; earlier long‐term data similarly estimated an SGA annual incidence near 0.8% in adults against approximately 5.4% for haloperidol. Two caveats temper these findings. First, much of the apparent prevalence reflects cumulative lifetime antipsychotic exposure rather than current drug class alone, as illustrated by the markedly lower prevalence in FGA‐naive patients (7.2%) than in SGA‐treated cohorts with prior FGA exposure (23.4%). Second, the evidence base is dominated by 1‐year horizons, severity was inconsistently reported, and signals of publication bias were detected for the TD outcome. Collectively, these data support preferential use of SGAs to reduce TD burden while underscoring the need for ongoing monitoring, attention to modifiable risk factors, and minimization of cumulative exposure across both drug classes.

**TABLE 3:** Systematic reviews and meta‐analyses of tardive dyskinesia risk with antipsychotic (neuroleptic) medications.

Study	Design	N	Population/scope	Key findings
Correll et al., 2004 (Am J Psychiatry)	Systematic review of 1‐year studies	11 studies; 2769 patients	Patients on SGAs > = 1 year (risperidone, olanzapine, quetiapine, amisulpride, ziprasidone); comparator haloperidol or placebo in 4 trials	Weighted mean annual TD incidence on SGAs: 0.8% (adults), 6.8% (mixed adult/elderly), 5.3% (age > = 54 year), 0% (children); haloperidol comparator 5.4%/year SGAs carry reduced TD risk vs. FGAs, though comparator haloperidol doses were relatively high.
O'Brien, 2016 (Int J Geriatr Psychiatry)	Systematic review & meta‐analysis	Older‐adult studies (1957‐Jan 2015)	Older adults treated with FGAs vs. SGAs; validated rating scale + research diagnostic criteria	FGA prevalence: mild TD 53% (95% CI 39.0–68.4), probable TD 38% (95% CI 25.9–50.3). FGA probable‐TD incidence 23% (1 year), 42% (2 year), 57% (3 year). SGA 1‐year incidence: probable TD 7% (95% CI 4.4–10.2), persistent TD 3% (95% CI 1.5–4.2).
Carbon et al., 2017 (J Clin Psychiatry)	Cross‐sectional meta‐analysis	41 studies; 11,493 patients	Contemporary FGA and/or SGA treatment; mean age 42.8 year; 77.1% schizophrenia‐spectrum	Global mean TD prevalence 25.3% (95% CI 22.7–28.1). Current SGA 20.7% (95% CI 16.6–25.4) vs. current FGA 30.0% (95% CI 26.4–33.8); *p* = 0.002. FGA‐naive arms 7.2% vs. 23.4% in SGA cohorts with likely prior FGA exposure. SGA vs. FGA risk ratio 0.80 (95% CI 0.67–0.95).
Carbon et al., 2018 (World Psychiatry)	Meta‐analysis of head‐to‐head RCTs	57 RCTs (32 FGA, 86 SGA arms)	Randomized comparative trials; SGAs vs. FGAs and SGAs vs. SGAs	Annualized TD incidence: FGA 6.5% (95% CI 5.3–7.8) vs. SGA 2.6% (95% CI 2.0–3.1). SGA vs. FGA: RR 0.47 (95% CI 0.39–0.57); annualized rate ratio 0.35 (95% CI 0.28–0.45); NNT 20. Olanzapine and aripiprazole showed an advantage over other non‐clozapine SGAs.
Ali et al., 2021 (PLoS ONE)	Meta‐analysis of observational studies	15 studies	Patients on antipsychotics; pooled extrapyramidal side‐effect prevalence (PROSPERO CRD42020175168)	Pooled TD prevalence 7% (95% CI 4–9). Parkinsonism 20% (95% CI 11–28); akathisia 11% (95% CI 6–17); any EPSE 31%–37%. Possible publication bias indicated for the TD estimate (Egger's test).

Abbreviations: CI, confidence interval; EPSE, extrapyramidal side effect; FGA, first‐generation antipsychotic; NNT, number needed to treat; RCT, randomized controlled trial; RR, risk ratio; SGA, second‐generation antipsychotic; TD, tardive dyskinesia.

Population estimates for MCP‐associated TD vary widely with reported risks around 0.1 per 1000 patient‐years overall, with higher risk among elderly females, individuals with diabetes, renal/hepatic impairment, or those co‐exposed to antipsychotics [6, 17]. When MCP is necessary beyond short courses, our data support considering intermittent or pulse regimens rather than continuous dosing.

This study's key strengths include the very large, nationally representative dataset with longitudinal follow‐up; more specific definition based on two documentations for TD definition to improve positive predictive value; and incidence and time‐to‐event analysis across all MCP users and in the subgroup with GP.

Domperidone, a D2 receptor antagonist available internationally and previously accessible in the US via FDA Expanded Access IND, is thought to carry a lower risk of TD than MCP owing to its avid P‐glycoprotein‐mediated efflux at the blood–brain barrier, which limits CNS penetration—with PET imaging confirming brain exposure approximately 2.4‐fold lower than MCP [18]. A systematic review and meta‐analysis of 28 gastroparesis studies found extrapyramidal events, including TD, were reported more frequently with MCP than domperidone [19]. A query of FAERS identified 81 reports of dyskinesia (including possible tardive dyskinesia) for domperidone (1.99% of all domperidone adverse event reports); however, FAERS does not distinguish tardive from acute drug‐induced dyskinesia at the coding level, and as domperidone was never FDA‐approved, the database substantially underrepresents its true adverse event exposure—particularly for patients who imported the drug. Case reports confirm that domperidone‐induced TD, while rare, can occur in patients with compromised blood–brain barriers [20], underscoring that its neurological safety advantage over MCP is relative, not absolute.

However, the study has limitations inherent to all retrospective claims analysis due to potential outcome misclassification, exposure misclassification as we lacked granular dose and days‐supply adherence metrics, and confounding by disease severity, since sicker GP patients may preferentially receive continuous MCP. Additionally, administrative claims data do not capture the level of clinical granularity required to distinguish diabetic gastroparesis with extrinsic (autonomic) or intrinsic (enteric) neurodegeneration from idiopathic gastroparesis without neurodegeneration—a distinction that would require physician clinical notes from electronic medical records, which are highly unlikely to provide accurate and extensive documentation of the presence or absence of those components of neurodegeneration, and was therefore beyond the scope of this study. Co‐exposures to other dopamine antagonists (e.g., antipsychotics, prochlorperazine, and promethazine) and neurologic comorbidities could not be fully controlled. Finally, this study was limited to oral metoclopramide and does not address nasal metoclopramide (Gimoti), which has been available for approximately 2 years. Whether nasal administration—given its closer proximity to the brain—confers a differential risk of TD through distinct neurological mechanisms remains an important and unanswered question that warrants dedicated future investigation.

Within the limitations inherent to real‐world claims data, our findings align with regulatory guidance and existing literature: in cases where maintenance MCP is unavoidable, intermittent dosing, combined with close monitoring and transition plans to alternative therapies may offer a practical approach to mitigating the risk of TD and delaying its onset in routine clinical settings.

## Author Contributions

Pierantonio Russo: Study conception and design; drafting of the manuscript. Ramaa Nathan: Study conception and design; data analysis and interpretation; drafting of the manuscript. Michael Camilleri: Critical revision of the manuscript for important intellectual content. All authors approved the final version of the manuscript for publication.

## Conflicts of Interest

Dr. Camilleri is an advisor for Evoke Pharma with payment for his services to Mayo Clinic not to himself. He has received research support from Vanda for clinical trial of tradipitant in GLP‐1‐induced nausea and vomiting. Dr. Russo and Dr. Nathan are employees of EVERSANA.

## Data Availability

The data that support the findings of this study are available on request from the corresponding author. The data are not publicly available due to privacy or ethical restrictions.
